# 3D Resin-coated pressure sensor response for bite force assessment: A pilot study

**DOI:** 10.34172/joddd.2023.37142

**Published:** 2023-07-17

**Authors:** Tânia Soares, Marco Marques, Cláudia Barbosa, Mário Vaz, Maria Helena Figueiral

**Affiliations:** ^1^Faculty of Dental Medicine, University of Porto, Porto, Portugal; ^2^LAETA Biomechanics Laboratory, INEGI. Faculty of Engineering, University of Porto, Porto, Portugal; ^3^Faculty of Health Sciences, University of Fernando Pessoa, Porto, Portugal; ^4^Department of Mechanical Engineering, Faculty of Engineering, University of Porto, Porto, Portugal; ^5^Department of Oral Rehabilitation, Faculty of Dental Medicine, University of Porto, Porto, Portugal

**Keywords:** Bruxism, Occlusal splint, Monitoring, Ambulatory, Bite force

## Abstract

**Background.:**

Occlusal splints with sensors help in the bruxism diagnosis and monitoring, by recording the patient’s bite force. The aim of this study was to evaluate the accuracy of a pressure sensor when it is covered with different thicknesses of a 3D printing resin (Anycubic 405nm Translucent Green UV Resin, Anycubic, UK).

**Methods.:**

In this preliminary study, the evaluated sensor (FlexiForce A201 Sensor, Tekscan) was firstly calibrated without any type of cover material, and later tested with 3D printing resin with different thicknesses (1 mm, 1.15 mm, 1.4 mm and 1.6 mm). The load tests were performed by a force tester (MultiTest 2.5 dV, Mecmesin).

**Results.:**

When the pressure sensor was covered with resin of 1mm and 1.6 mm thick specimens, a higher difference was found between the applied load and the corresponding sensor reading.

**Conclusion.:**

It was concluded that it is possible to use this type of pressure sensor and that it showed better accuracy with the 1.15 mm and 1.4 mm 3D printing resin covering.

## Introduction

 Sleep bruxism is classified as an activity of the masticatory muscles, rhythmic or non-rhythmic, with teeth grinding or clenching during sleep.^1,2^ The diagnosis of bruxism is based on self-reports and clinical signs, such as noises associated with grinding, muscle fatigue on waking and tooth wear. Polysomnography is the gold standard for diagnosing bruxism, but it is not widely used because it is an expensive procedure and must be performed in a hospital environment.^3^

 Currently, there is no consensus on the most effective treatment for bruxism, with the suggestion of symptom relief through intraoral devices, pharmacotherapy, behavioral strategies and physical therapy.^4^ Thus, occlusal splints are used to prevent tooth wear and help in muscle relaxation.^5^ Some studies report the use of occlusal splints with the inclusion of pressure sensors in order to quantify the bite force and consequently help diagnose bruxism.^6^ However, despite the diversity of devices described, one of the limitations reported is related to its volume, resulting from the inclusion of sensors and electronic devices necessary for its operation, which makes the splint uncomfortable for the patient.^7^ Thus, it is important to understand which are the ideal conditions for the inclusion of the sensor, namely the maximum thickness of the material that can cover the sensor and, at the same time, allow a correct reading. To answer this question, the aim of this study was to evaluate the load sensitivity of a pressure sensor when it is embedded in a splint and covered with different thicknesses of a 3D printing resin.

## Methods

**Figure 1:**
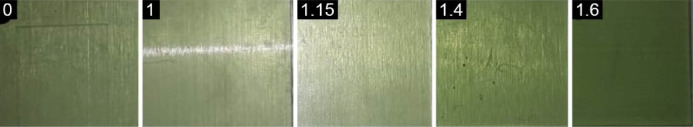
Base and cover specimens with different thicknesses (1 mm, 1.15 mm, 1.4 mm and 1.6 mm)

 First the 3D printing resin specimens (Anycubic 405nm Translucent Green UV Resin, Anycubic, UK) were printed by the LCD technique on the Phrozen Sonic Mighty 4K 3D Printer (Phrozen, Taiwa). One specimen (10 mm × 10 mm × 1 mm) was printed (Figure 1) with a small cavity (0,15mm of thickness) to insert and fix the sensor during all tests. This one was called the “base specimen”. The other four test specimens were printed with the same dimensions (10 mm × 10 mm) but with different thicknesses (1 mm, 1.15 mm, 1.4 mm and 1.6 mm) and were placed alternately over the “specimen base” with the pressure sensor to perform the load tests.

 The load tests were performed on a force tester (MultiTest 2.5 dV, Mecmesin). First, the pressure sensor (FlexiForce A201 Sensor, Tekscan) was calibrated by applying loads (5, 20, and 30 Newtons (N)) directly over the sensor included in the “base specimen”. It was confirmed if the values were the same in the MultiTest and in the sensor software.

**Figure 2:**
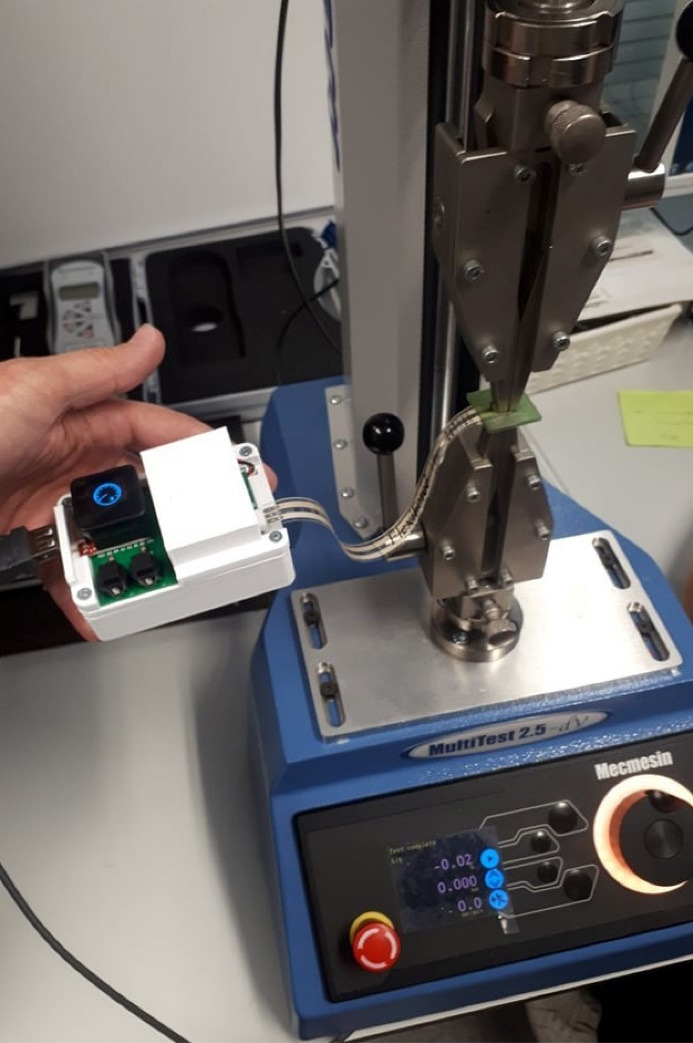
Sensor included between samples in MultiTest machine for load testing

 After this calibration, the tests were performed with the sensor interposed between the “base specimen” and the different thickness 3D printing resin specimens. A preload of 1N was applied in all tests to standardize the initial conditions for all specimens. Thus, displacements of 60 millimetres per minute (mm/min) were applied, with cycles of 15 repetitions, which allowed reaching loads of about 20N (Figure 2), to evaluate the difference between the value measured by the sensor and the known load applied by the MultiTest.

## Results

 With this test, the applied load and corresponding sensor reading values were obtained for each of the test specimens. The applied load was registered by the sensor differently according to the thickness of the specimen. The percentage of difference between these two parameters was calculated, and it was found that the 1.15 mm and 1.4 mm specimens have a lower percentage of difference (Table 1).

**Table 1:** Percent of difference between sensor reading and applied load, per specimen

	**Specimen ** **1 mm**	**Specimen 1.15 mm**	**Specimen ** **1.4 mm**	**Specimen ** **1.6 mm**
Sensor reading	11.95	18.11	18.92	12.96
Applied load	19.32	19.84	21.16	20.74
% Difference	38.15	8.71	10.58	37.52

## Discussion

 There are some studies describing the inclusion of pressure sensors in a splint.^6,8^ However, there are no reports in the literature on the incorporation of sensors in splints made by 3D printing.

 Some authors, who have developed studies with the inclusion of pressure sensors in occlusal splints, stated that sensors should be located approximately 1mm below the occlusal surface, and therefore the sensor is covered by 1 mm of thickness of the splint material.^9,10^ In this work, the pressure sensor covered with 1.15 mm or 1.4 mm specimens was found to have the best response. For a clinical point of view this could be important, not only for sensor accuracy, but also because these thicknesses may be well tolerated, as they do not lead to an occlusal vertical dimension increase, which could be considered harmful for the majority of the patients.^11^

 Kinjo et al^12^ used a pressure sensor like the one presented in this article (Flexi Force A301-25, Tekscan Inc., South Boston, MA, USA), but covered with a resilient material, ethylene vinyl acetate. The authors tested the pressure sensor between specimens with 2 mm thick and 30 mm in diameter, applying load at a rate of 0.25 mm/min. The authors concluded that, in this configuration, it was only possible to reliably measure forces up to 70N, forces much higher than those used in this study, so not only the thickness but also nature and modulus of elasticity of the material could affect the performance of this kind of pressure sensor.^13^

 Kim et al^13^ performed an experimental test to calibrate the pressure sensor they developed for placement in an occlusal splint. They initially tested the sensor between two metal plates and applied 0.5 mm of compression at a speed of 1.0 mm/min, 5 times, with a Zwick instrument to test the sensor response. Subsequently, they used polymethylmethacrylate in different thicknesses (0.8 to 1.4 mm) to cover the sensor and found that the acrylic cover affects the sensitivity of the pressure sensor, because the applied load induces a bending in the acrylic, which may result in incorrect detection of the applied load. The thicker the layer covering the sensor, the greater the force required to achieve the same load on the sensor.

 Aoki et al^14^ incorporated pressure sensors in an occlusal splint and found as a limitation of their work the difficulty in controlling the material thickness covering the sensor and how it interferes with sensor accuracy. The conclusions of these authors corroborate the results of this work that presents differences between the sensor reading and the load applied with different thicknesses of covering material.

## Conclusion

 It can be concluded from this study, under these investigation conditions, that this type of sensor covered by a 3D printing resin is sensible to different applied loads. It was also found that the thickness of the resin on the sensor influences its accuracy, with resin thicknesses of 1.15mm and 1.4 mm showing better results.

## Acknowledgments

 Rita Rynkevic for her help to development the experimental procedures.

## Competing Interests

 Not applicable.

## Ethical Approval

 Not applicable.

## Funding

 The authors received no financial support, all costs were supported by the authors.
